# Editorial: Food of the future: underutilized foods

**DOI:** 10.3389/fnut.2023.1307856

**Published:** 2023-11-07

**Authors:** Salej Sood, Nikhil Malhotra, Kuldeep Tripathi, Natalie Laibach, Amparo Rosero

**Affiliations:** ^1^Indian Council of Agricultural Research-Central Potato Research Institute, Shimla, Himachal Pradesh, India; ^2^Indian Council of Agricultural Research-National Bureau of Plant Genetic Resources Regional Station, Shimla, Himachal Pradesh, India; ^3^Indian Council of Agricultural Research-National Bureau of Plant Genetic Resources, New Delhi, India; ^4^Centre for Research on Agricultural Genomics, Barcelona, Spain; ^5^Colombian Corporation for Agricultural Research, Mosquera, Colombia

**Keywords:** climate resilience, crop diversity, healthy foods, orphan crops, nutritional significance, qualitative food security

The world faces an essential challenge of a looming food crisis, exacerbated by the impending surge toward a projected global population of close to 10 billion by the year 2050, escalating an increasing burden on our current food systems. At the same time, agriculture impacts biodiversity and consumes the valuable resource land to critical extents. Climate change and shortage of natural resources become unprecedented challenges for agro-food value chains around the world. Given these challenges, crop diversification could be a strategy to guarantee our future food supply in a sustainable way, then, we must go beyond major and well-known crops. Moreover, it has been demonstrated that dietary preferences and choices have a direct impact on our health. Vegetarian and vegan alternatives are becoming more prevalent, and much emphasis has been placed on following a plant-based diet and avoiding items derived from animals. The main issue with the current agriculture and food systems revolves around emphasizing a limited set of staple crops, mainly as sources of energy-dense foods but poor-quality nutrition (1). About 60% of global dietary energy consumption comes from three major crops viz. rice, maize, and wheat. Dependence on a few staple crops puts food security at risk, especially under climate change scenarios. Vulnerable populations, including smallholder farmers, children, women, and indigenous communities relying on traditional crops, may face threats to their livelihoods and food security (2), due to crop genetic erosion caused by underutilization of these crop species. Underutilized crops may promote better health for both individuals and the environment due to exceptional adaptation traits and nutritionally superior profiles compared to major crops (3–6). Therefore, the use of neglected and underutilized species into the current cropping systems could help reduce imbalanced diets, food scarcity and diversify the homogeneous crops systems. Many edible plant species are still underutilized, but they have the potential to drastically reduce the overreliance on a small number of staple crops in conventional agriculture (7). By incorporating so-called orphan crops into our existing crop portfolio along with other measures of sustainable agriculture and agrobiodiversity required for environmentally friendly farming, sufficient supply with highly nutritional food may be secured while mitigating the consequences of environmental change.

This particular topic covered crop diversity, nutritional importance, breeding and omics approaches, as well as physiological and molecular responses to environmental cues of different underutilized crops. The Himalayan region is one of the biodiversity hotspots and home to several medicinal plants with unique health-promoting properties (3). An article by Thakur et al. presented the beneficial properties, i.e., health benefits and therapeutic advantages of five edible plants, *Allium rubellum, Berberis chitria, Capsella bursa-pastoris, Stellaria aquatica, and Rheum emodi*, consumed by the tribal Gaddi community in Himachal Pradesh.

During times of drought and acute poverty, orphan legumes provide food and nutritional security to rural people with limited resources, saving millions of lives. The increase in their demand can be attributed to the growing global inclination toward transitioning from a diet rich in animal protein to one characterized by a higher content of vegetarian protein. Underutilized legumes have substantial opportunities to enhance food security, meet dietary needs, and advance agriculture under environmental constraints (8). Future foods are not yet incorporated into major breeding programs but interest is increasing, given their benefits. However, various methods involving breeding and biotechnological approaches are available for the genetic improvement of underutilized crops to assist farmers in diversifying agricultural systems and increasing profitability. Samal et al. emphasized the nutritional significance of underutilized legumes and advanced breeding methodologies for their qualitative and quantitative improvement. Likewise, rice bean (*Vigna umbellata*), a lesser-known legume species in the *Vigna* genus that has gained interest in recent years as a crop promoting food and nutritional security, was highlighted by Katoch et al. for its nutritional and nutraceutical benefits.

Underutilized tuber crops play an important role in ensuring food security in developing countries due to their high energy value and carbohydrate content. Among them, *Dioscorea* is an important genus characterized by large underground tubers and aerial bulbils. It nourishes millions of people worldwide, especially in the tropics and subtropics (9). Chinese yam additionally has multiple health benefits and is also cultivable in temperate climates (10) though till now scarcely incorporated in molecular biology research. RNA sequencing analysis of two tuber shape variants of *D. polystachya* by Riekötter et al. established the significance of brassinosteroids in tuber shape, offering insights into the role of plant hormones in the development of yam storage organs. They elucidated that brassinosteroids can influence tuber shape in Chinese yam by modulating gene expression in cell growth. The information generated could be useful for breeding strategies and to improve crop systems, promoting the use of this crop under commercial schemes.

Due to their superior nutrition, essential minerals, amino acids, and gluten-free status, amaranth and quinoa have become popular pseudocereals and important substitutes for traditional cereals (11). These crops may benefit developing nations engaged in subsistence agriculture with limited agricultural resources. Despite the enormous genetic variability of these crops, the development of improved high-yielding varieties is slow. Hence, Anuradha et al. reviewed critical nutrition quality parameters, morphological descriptors, breeding behavior, accessible genetic resources, and breeding methodologies for amaranth and quinoa to shed light on future breeding strategies to generate superior genotypes. Another important medicinal plant covered in this topic is Perilla, an annual aromatic plant of the Lamiaceae family, widely cultivated and consumed in the most Asian countries. Aochen et al. reviewed Perilla's nutritional and medicinal properties and presented future insights into the research and development for its improvement.

Underutilized crops have the potential to be crucial in resolving the world's ongoing food challenges. They offer sustainability, adaptability, and nutritional value that can contribute to food security, agrobiodiversity conservation, and a more resilient agricultural system and agro-food value chains. Thousands of traditional or underutilized crops exist, but only a select number have garnered much attention. Embracing these crops is a step toward a more resilient, sustainable and diverse global food system. These crops plants are significant not just for conservation, but also for gaining insights into their genetic background and important genes or alleles that favor their survival in harsh environments (12). Limited progress has been made in developing nutritionally superior underutilized crops; thus, increased endeavors are essential to integrate these crops into mainstream agriculture.

## Author contributions

SS: Conceptualization, Writing—original draft. NM: Writing—review & editing. KT: Writing—review & editing. NL: Writing—review & editing. AR: Writing—review & editing.
